# DNA methylation regulates the secondary metabolism of saponins to improve the adaptability of *Eleutherococcus senticosus* during drought stress

**DOI:** 10.1186/s12864-024-10237-x

**Published:** 2024-04-02

**Authors:** Shuo Wang, XueLei Zhao, Chang Li, Jing Dong, JiaCheng Ma, YueHong Long, ZhaoBin Xing

**Affiliations:** College of Life Sciences, North China University of Science and Technology, Tangshan, China

**Keywords:** Drought stress, DNA methylation, Transcription factor, *Eleutherococcus senticosus*, Secondary metabolite, Saponin

## Abstract

Plant growth and development can be significantly impacted by drought stress. Plants will adjust the synthesis and accumulation of secondary metabolites to improve survival in times of water constraint. Simultaneously, drought stress can lead to modifications in the DNA methylation status of plants, and these modifications can directly impact gene expression and product synthesis by changing the DNA methylation status of functional genes involved in secondary metabolite synthesis. However, further research is needed to fully understand the extent to which DNA methylation modifies the content of secondary metabolites to mediate plants’ responses to drought stress, as well as the underlying mechanisms involved. Our study found that in *Eleutherococcus senticosus* (*E. senticosus*), moderate water deprivation significantly decreased DNA methylation levels throughout the genome and at the promoters of *EsFPS*, *EsSS*, and *EsSE*. Transcription factors like *Es*MYB-r1, previously inhibited by DNA methylation, can re-bind to the *EsFPS* promotor region following DNA demethylation. This process promotes gene expression and, ultimately, saponin synthesis and accumulation. The increased saponin levels in *E. senticosus* acted as antioxidants, enhancing the plant’s adaptability to drought stress.

**Supplementary Information:**

The online version contains supplementary material available at 10.1186/s12864-024-10237-x.

## Introduction

According to information from the sixth assessment report of the Intergovernmental Panel on Climate Change, there has been a significant 1.5°C increase in global temperature from the pre-industrial era [1]. As a result of the temperature rise, some areas have experienced more frequent and severe droughts, rendering the plants that grow those areas vulnerable to drought conditions. Drought stress negatively affects many organisms, with plants being the most vulnerable [2]. Additionally, drought has significantly reduced the global production of medicinal plants in several regions worldwide [3]. Therefore, it has become crucial to research how plants adapt to dry conditions under drought stress.

Drought stress disrupts several physio-biochemical processes, hindering the growth and development of plants [4]. While their overall biomass and productivity would decrease significantly, plants can often withstand water scarcity to some extent [5]. Drought stress can interrupt photosynthesis, growth, and other physiological and biochemical processes [6]. Over the past 30 years, researchers have thoroughly studied the molecular and cellular mechanisms of plant drought responses [5]. Plants use two coping mechanisms when stressed by drought. One is the avoidant plant, which, when faced with a water deficit, accelerates its metabolism, increases the amount of water and nutrient absorption, and reduces the synthesis of secondary metabolites. The other type of plant adapts to dry conditions by altering its osmotic pressure, changing its cell wall properties, and producing more antioxidants and secondary metabolites [7–8]. Nevertheless, in both types of plants, the synthesis rate of secondary metabolites increases under prolonged drought stress. These metabolites act as antioxidants, helping the plant scavenge reactive oxygen species formed in the body due to oxidative stress induced by drought stress, thereby reducing the stress on plant cells [7]. Triterpenoids exhibit antioxidant activity and play a crucial role in scavenging reactive oxygen species, as demonstrated by studies conducted on medicinal plants such as *Glycyrrhiza glabra* L. [9].

Research has revealed that plants alter their physiological metabolism in addition to epigenetic modification of DNA methylation to improve their response to drought environmental conditions [10]. Due to this alteration, plants can better withstand external environmental stress, such as water shortage. Methyl (CH_3_) must bind with a phosphodiester bond to the fifth carbon atom of the cytosine-guanine dinucleotide (CpG) to form 5-methylcytosine (5-mC). This is known as DNA methylation. This alteration significantly impacts the epigenetic regulation of eukaryotic cell genomes [11]. Plants under drought stress have been shown to change their DNA methylation sites and patterns. These changes are specific to particular stress sites and types [12]. For example, drought stress altered the DNA methylation status of 2.48% of the genome in *Gossypium hirsutum* L. [13]. Similarly, in *Malus prunifolia* Borkh., the promoter region of the dehydration-responsive element binding proteins gene decreased in DNA methylation from 60% to 25%, resulting in a more than 100-fold increase in expression [14]. These findings demonstrate the critical role that DNA methylation plays as an epigenetic modification in plants’ response to drought stress [10].

Modifications in the DNA methylation state of the plant secondary metabolite synthase gene promoter can directly impact the expression of these genes and the synthesis of related secondary metabolites. The secondary metabolism of *Salvia miltiorrhiza* is regulated by the transcription of *SmGPPS*, which is influenced by DNA methylation of genes like geranyl pyrophosphate synthase (GPPS), involved in the synthesis of tanshinone and salvianolic acid [15]. However, it is yet unknown how precisely modifications in secondary metabolism relate to the state of DNA methylation in plants under drought stress.

*Eleutherococcus senticosus* (*E. senticosus*) (Rupr. et Maxim) Maxim, sometimes called Siberian ginseng and a member of the Araliaceae family, is an invaluable medicinal plant [16]. Its primary active components, triterpenoid saponins, are critical metrics for assessing its therapeutic quality [17–19]. Recent studies on *E. senticosus* show the plant thrives in moist soil environments for growth and photosynthesis, and it accumulates secondary metabolites, such as triterpenoid saponins, under moderate drought stress [20]. Studies on other medicinal plants have shown similar results, with mild drought stress favoring the synthesis and build-up of secondary metabolites in plants [21]. Drought stress, for instance, increased *Dendrobium moniliforme* (L.) Sw. synthesizing secondary metabolites enhances medicinal plants’ quality [22]. In our earlier research, we found a negative correlation between the number of saponins and the DNA methylation ratios of the promoters of the crucial enzyme genes for triterpenoid saponin synthesis, namely, farnesyl diphosphate synthase (*EsFPS*), squalene synthase (*EsSS*), and squalene epoxidase (*EsSE*). Furthermore, site-specific DNA methylation significantly impacted gene expression, with secondary metabolism being more significant than the DNA methylation ratio [18]. The findings above indicate that DNA methylation involved in saponin synthesis can directly impact the level of secondary metabolites that accumulate [18–19]. Therefore, it is crucial to investigate how drought stress affects the DNA methylation state of *E. senticosus* and regulates saponin metabolism to improve the resistance of plants to drought stress and better understand the mechanisms by which plants adapt to drought.

## Materials and methods

### Experimental materials

A group of 50 two-year-old *E. senticosus* from the same clonal line that cuttings had propagated were divided into five groups and raised in the plant culture room of the North China University of Science and Technology at a temperature of 24℃, with 16 h of light and 8 h of darkness. Professor ZhaoBin Xing has identified the materials used from the School of Life Sciences at North China University of Science and Technology as *Eleutherococcus senticosus*, a plant of the Araliaceae family. The voucher specifications were stored in the School of Life Sciences, North China University of Science and Technology laboratory. Based on the soil weight, the soil water content was controlled to be 30%, 50%, 70%, and 90%, respectively. Simultaneously, the DNA demethylation reagent 5-Azacytidine (5-AzaC) was used to treat *E. senticosus* and lower its DNA methylation levels to understand better the effects of various DNA methylation states on secondary metabolism. After maintaining each water content level for 30 days, the leaves of *E. senticosus* were harvested for further examination.

### Experimental methods

#### Extraction and transcriptome sequencing of the total RNA of *E. senticosus*

The RNAprep Pure Plant Plus Kit (Tiangen, Beijing, China) extracted the RNA of *E. senticosus*. The input material for the RNA sample preparations was 1 µg of total RNA per sample. Following the manufacturer’s instructions, sequencing libraries were created using the NEBNext UltraTM RNA Library Prep Kit for Illumina (NEB, USA), and index codes were added to each sample to identify its sequences. In short, poly-T oligo-attached magnetic beads separated mRNA from total RNA. Divalent cations performed fragmentation in NEBNext First Strand Synthesis Reaction Buffer (5×) at high temperatures. A random hexamer primer and M-MuLV Reverse Transcriptase (RNase H) were used to create first-strand cDNA. RNase H and DNA Polymerase I were then used to synthesize second-strand cDNA. Exonuclease/polymerase activities turned the remaining overhangs into blunt ends. To prepare for hybridization, the NEBNext Adaptor with a hairpin loop structure was ligated after the 3′ ends of DNA fragments had been adenylated. AMPure XP system (Beckman Coulter, Beverly, USA) was used to purify the library fragments to select cDNA fragments that were preferably 250–300 bp in length. Then, size-selected, adaptor-ligated cDNA was treated with 3 µL USER Enzyme (NEB, USA) at 37°C for 15 min and then heated to 95°C for 5 min before PCR. Next, Phusion high-fidelity DNA polymerase, universal PCR primers, and index (X) primers were used for PCR. Finally, PCR products were purified (AMPure XP system), and the library quality was evaluated on the Agilent Bioanalyzer 2100 system. According to the manufacturer’s instructions, the index-coded samples were clustered using a cBot Cluster Generation System using the TruSeq PE Cluster Kit v3-cBot-HS (Illumina). Following cluster formation, 125 bp/150 bp paired-end reads were produced by sequencing the library preparations on an Illumina Hiseq platform. Fastp (v0.19.3) filtered the original data to exclude adapters and remove paired reads if the N content in any sequencing read exceeded 10% of the base number of reads. A pair of reads would be eliminated if the percentage of low-quality (Q ≤ 20) bases in the reads exceeded 50%. Clean reads served as the basis for all subsequent analyses. Trinity (v2.11.0) was used for transcriptome assembly. Relevant transcripts were gathered into “gene” clusters using a corset (https://github.com/trinityrnaseq/trinityrnaseq) (Accession: SRX13417593-SRX13417601). Using DIAMOND BLASTX software, the unigene sequence was compared with databases from KEGG, NR, Swiss-Prot, Gene Ontology (GO), COG/KOG, and Trembl. The unigene was created by splicing, assembling, and hierarchical clustering of high-quality data. Using HMMER software, the annotation information for unigene was retrieved by comparing it with the Pfam database after predicting the amino acid sequence [19, 23].

#### Extraction and analysis of metabolites of *E. senticosus*

A method based on the literature was used to extract the metabolites of *E. senticosus* [18–19]. For *E. senticosus*, the metabolome analysis was carried out using a UPLC-ESI-MS/MS system (UPLC, SHIMADZU Nexera X2, https://www.shimadzu.com.cn/; MS, Applied Biosystems 4500 Q TRAP, https://www.thermofisher.cn/cn/zh/home/brands/applied-biosystems.html). The following were the analytical conditions Agilent SB-C18 (1.8 μm, 2.1 mm × 100 mm) column for UPLC: solvent A, pure water with 0.1% formic acid, and solvent B, acetonitrile with 0.1% formic acid, made up the mobile phase. Sample measurements were performed using a gradient program with 95% A and 5% B as the starting conditions. A linear gradient to 5% A, 95% B was programmed within 9 min, and a composition of 5% A, 95% B was maintained for 1 min. After 1.1 min, a composition of 95% A and 5% B was adjusted within 1.1 min and maintained for 2.9 min. The column oven was set to 40°C, the injection volume was 4 µL, and the flow velocity was set at 0.35 mL per minute. An alternate connection for the effluent was made to an ESI-triple quadrupole-linear ion trap (QTRAP)-MS. LIT and triple quadrupole (QQQ) scans were obtained using an AB4500 Q TRAP UPLC/MS/MS System equipped with an ESI Turbo Ion-Spray interface and operated in positive and negative ion modes. The system was managed by Analyst 1.6.3 software (AB Sciex). The following were the ESI source operation parameters: ion source, turbo spray; source temperature 550°C; ion spray voltage (IS) 5500 V (positive ion mode)/−4500 V (negative ion mode); ion source gas I (GSI), gas II(GSII), and curtain gas (CUR) were set at 50, 60, and 25.0 psi, respectively; and the collision-activated dissociation (CAD) was high. In QQQ and LIT modes, 10 and 100 µmol/L polypropylene glycol solutions were used for instrument tuning and mass calibration. As part of MRM experiments, QQQ scans were obtained using medium collision gas (nitrogen). Additional DP and CE optimization were carried out for individual MRM transitions. Depending on the metabolites that eluted at each interval, a particular set of MRM transitions was monitored.

#### Determination of the total saponin content of *E. senticosus*

Using the literature as a guide, the total saponin content of *E. senticosus* was extracted [19]. Accurately weigh 2.6 mg of oleanolic acid standards (Solarbio, China) and place them in a 10 mL volumetric flask. Dissolve the oleanolic acid standards in methanol up to the mark and shake well. Precisely extract 1, 2, 4, 8, and 10 µL from the solution, dissolve each volume in methanol up to the mark in separate 10 mL volumetric flasks, then shake well. Filter the samples using a microporous filter membrane (0.22 μm pore size) and prepare a standard solution of oleanolic acid in the injection bottle. High-Performance Liquid Chromatography (HPLC) was used to determine the peak areas of various concentrations of oleanolic acid standards. A Kromasil 100-5 C18 column 250 × 4.6 mm (Kromasil, Sweden) was used for the chromatography, which was carried out on an Essentia LC-16 (Shimadzu, Japan) at 35℃ column temperature, 0.5 mL/min methanol mobile phase flow rate, and 15 min of 210 nm detection wavelength. HPLC determined the peak areas of different concentrations of oleanolic acid standards and was used to calculate the peak areas of various concentrations of oleanolic acid standards. From there, the standard curve *y* (µg/g) = 0.65 (*x* − 2500.9)/4238.4*t* (where *y* is the oleanolic acid content in units of µg/g, *x* is the peak area, and *t* is the fresh weight of the sample in units of g) was built. The oleanolic acid content was then used to replace the total saponin content of the sample [18].

#### Detection of the DNA methylation status of *E. senticosus*

The Plant Genomic DNA Extraction Kit (Tiangen, Beijing, China) was utilized to extract the genomic DNA of *E. senticosus*. The samples were tested for the genomic DNA methylation ratio following the guidelines provided by the MethylFlash Global DNA Methylation (5-mC) Enzyme-linked immunosorbent assay Easy Kit (Colorimetric) (Epigentek, USA). The DNA Bisulfite Transformation Kit (Tiangen, Beijing, China) was used to assess the DNA methylation of the promoters of the *EsFPS*, *EsSS*, and *EsSE* genes [18]. Supplementary Table 1 lists the primers used in this study.

#### Screening of differentially expressed genes, transcription factors, and metabolites

DESeq software was utilized to assess the FPKM values of the genes. Fold change (FC) and test probability (FDR) were used to filter for differentially expressed genes, with the criterion being FDR < 0.01 and|log^2^ FC| ≥1. To screen for differential metabolites, the Fold Change and variable importance projection (VIP) value were combined; the screening criteria were|log^2^ FC| ≥1 and VIP ≥ 1. The iTAK program predicted transcription factors [24–25]. The transcription factors with the smallest *P*-value of significant expression differences were chosen for further tests based on the size of the *P*-value. During the screening process for differential metabolites based on grouping information, such as 50% vs 5-AzaC, the upregulation of differential genes or metabolites suggests that the relative content of the gene or metabolite is low in the 50% water treatment group and high in the 5-AzaC treatment group. Conversely, the downregulation of differential genes or metabolites indicates that the gene or metabolite has a relatively high content in the 50% water treatment group and a relatively low content in the 5-AzaC treatment group.

#### Correlation and expression level analysis between different omics

A free online data analysis platform (https://cloud.metware.cn), differential gene, metabolite, and transcription factor expression analysis heatmaps and correlation analysis maps were detected in transcriptome and metabolome sequencing data using the Metware Cloud. Determine the correlation between the methylation ratios of *EsFPS*, *EsSS*, and *EsSE* promoters determined by the bisulfite sequencing method and the FPKM gene expression values in transcriptome data. Evaluate the impact of *EsFPS*, *EsSS*, and *EsSE* promoter methylation on gene expression using the Pearson correlation calculation method.

#### Cloning and subcellular localization of transcription factors

The open reading frame (ORF) of every sensitive transcription factor was cloned based on the sequences of the sensitive transcription factor unigene in the transcriptome sequencing data. Using the WoLF PSORT website (https://wolfpsort.hgc.jp), the subcellular localization information of the screened sensitive transcription factor proteins was predicted. *Agrobacterium tumefaciens* GV3101 (Biomed, Beijing, China) was created using the PHG-sensitive transcription factor- green fluorescent protein (GFP) recombinant plasmid, and it was then invaded into *Allium cepa* L. epidermal cells. Using laser confocal scanning microscopy (Leica, Germany), the subcellular localization of each transcription factor was ascertained by locating the GFP fluorescence signal [26].

#### Electrophoretic mobility shift assay (EMSA) for transcription factor-DNA binding analysis

Using the Seamless Cloning Kit (Beyotime, Shanghai, China), the ORFs of sensitive transcription factors were ligated into the pGEX-4T-3 vector, and then *Escherichia coli* BL21 receptor cells were transformed. Every sensitive transcription factor has its expression induced [24]. The GST-tag Protein Purification Kit (Beyotime, Shanghai, China) was utilized to purify every sensitive transcription factor protein in preparation for EMSA analysis [27]. Amplifying the promoter sequences of the *EsFPS*, *EsSS*, and *EsSE* genes using PCR [18]. Following the recovery of promoter DNA sequences, CpG Methyltransferase (M.*Sss*I) (New England Biolabs, Beijing, China) was used to induce DNA methylation, and the resultant probes were utilized for EMSA analysis. The EMSA Probe Biotin Labeling Kit (Beyotime, Shanghai, China) biotin-labeled every sensitive transcription factor. The binding reaction contained 1 µL of 10X binding buffer, 2 µg of sensitive transcription factor proteins, and 1 µL of biotin-labeled *EsFPS*, *EsSS*, and *EsSE* promoter probes. The membranes were transferred to Amersham Hybond-N^+^ nylon membranes following 4% non-denaturing polyacrylamide gen electrophoresis. The presence or absence of the bands following development and fixing was used to determine the binding of each sensitive transcription factor to DNA [28].

#### Molecular docking of sensitive transcription factors with *EsFPS*, *EsSS*, and *EsSE* promoters

Sensitive transcription factors’ putative binding locations to the *EsFPS* promoter were identified through the JASPAR website (https://jaspar.genereg.net/). Sensitive transcription factors: protein homology modeling with the SWISS-MODLE website (http://www.swissmodel.expasy.org/interactive). Molecular docking of sensitive transcription factors with the *EsFPS* promoter was carried out in the literature [26]. PyMOL software was used for the visualization process.

#### Overexpression analysis of sensitive transcription factors

The pCAMIBIA1300-EGFP-MCS vector was ligated with the ORFs of sensitive transcription factors using the Seamless Cloning Kit (Beyotime, Shanghai, China). *A. tumefaciens* GV3101 receptor cells transformation. Segments 1 cm × 1 cm were cut from fresh mature *E. senticosus* leaves. A solution containing 100 µmol/L acetosyringone, 10 mmol/L MES, and 50 mmol/L MgCl_2_ was added to the converted *A. tumefaciens* GV3101, and the OD_600_ value was measured to be 0.6. After 10 min of 400 Pa vacuum filtrations, the chopped *E. senticosus* leaves were submerged in the above liquid. After spreading sterile three-layer filter paper onto a sterile petri dish and moistening it with sterile water, the filtered leaves were cultured for 72 h at 23°C. Using reverse transcription into cDNA from isolated RNA. Real-time PCR was used to identify the expression of sensitive transcription factors and *EsFPS*, *EsSS*, and *EsSE* [18]. Table 1 displays the primers that were utilized. Using HPLC, the amount of oleanolic acid in the leaves was ascertained.

## Results

### Effect of drought stress on DNA methylation level of *E. senticosus*

**Fig. 1:**
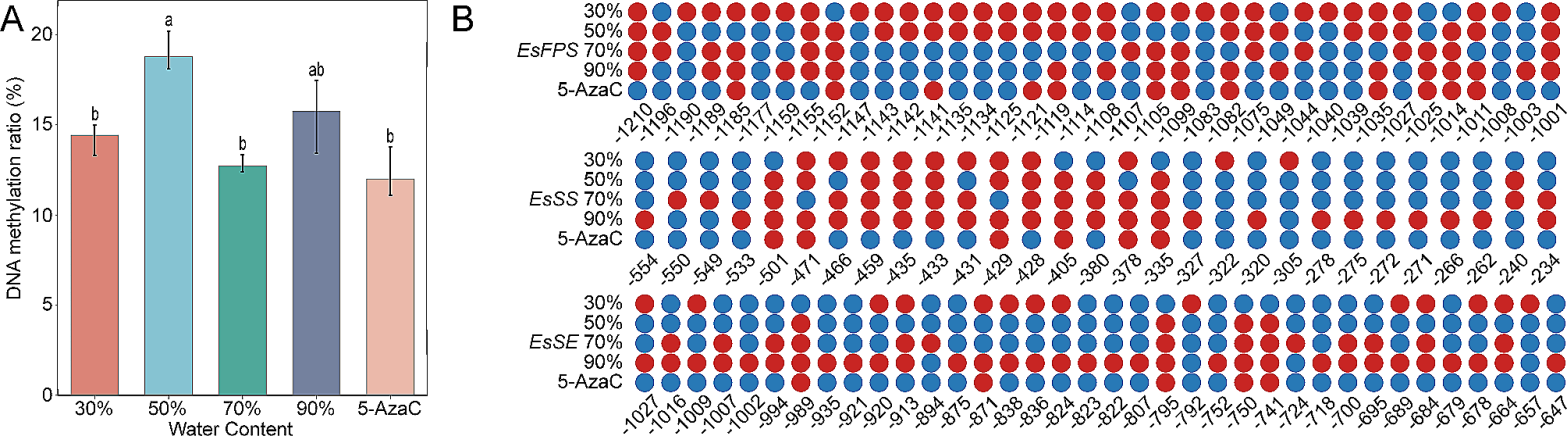
Effect of drought stress on the DNA methylation level of *E. senticosus*. **A**: DNA methylation ratios of the overall genomic DNA of *E. senticosus*; **B**: DNA methylation status of the promoters of *EsFPS*, *EsSS*, and *EsSE*. Note: lowercase letters indicate *P* < 0.05; blue circled dots indicate areas where DNA methylation did not occur; red circled dots represent sites where DNA methylation occurred; and numbers represent the distance (bp) from the start codon

The total genomic DNA methylation ratios of *E. senticosus* during drought stress are shown in Fig. 1. The highest genomic DNA methylation ratio (18.80%) was recorded at 50% water content. In comparison, the lowest genomic DNA methylation ratio (12.00%) was registered under the 5-AzaC treatment. The genomic DNA methylation ratios of *E. senticosus* decreased at 90%, 30%, and 70% water content (15.76%, 14.42%, and 12.73%, respectively). Resulfite sequencing analysis of the DNA methylation sites of the *EsFPS*, *EsSS*, and *EsSE* promoters revealed that, in 30–90% water content and 5-AzaC-treated *E. senticosus*, the DNA methylation ratios of the *EsFPS* promoter were 81.58%, 60.53%, 39.47%, 47.37%, and 31.58%. The DNA methylation ratios of the *EsSS* promoter were 37.93%, 37.93%, 51.72%, 82.76%, and 20.69%. The DNA methylation ratios of the *EsSE* promoter were 38.89%, 11.11%, 38.89%, 88.89%, and 13.89%. The 5-AzaC treatment group displayed the lowest DNA methylation among the *EsFPS* and *EsSE* promoters, with methylation ratios of 31.58% and 20.69%, respectively. At 50% water content, the methylation ratio (11.11%) of the *EsSE* promoter was the lowest.

### Effect of drought stress on the metabolites of *E. senticosus*

**Fig. 2:**
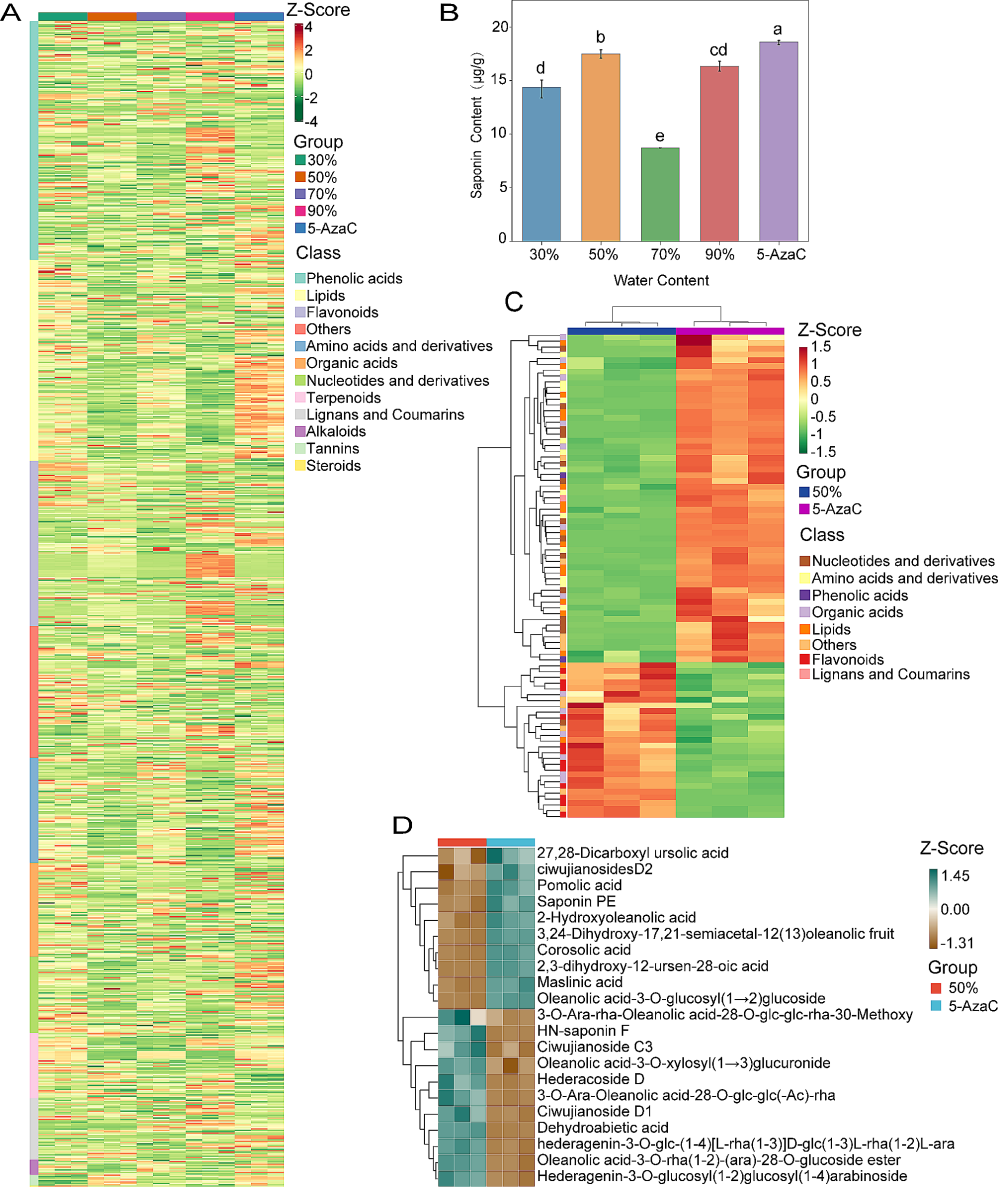
Effect of drought stress on metabolites of *E. senticosus*. **A**: Variation in *E. senticosus* overall metabolite levels under various drought stress; **B**: Variations in *E. senticosus* total saponin content under different drought stress; **C**: Variations in metabolites between groups of 50% vs 5-AzaC; and D: Variation in terpenoids metabolites between groups of 50% vs 5-AzaC. Note: lowercase letters indicate *P* < 0.05

A total of 857 metabolites were found in *E. senticosus* under drought stress treatment using the UPLC-MS/MS approach (Supplementary Table 2). Figure 2A depicts the metabolite changes of *E. senticosus* under various drought stress conditions. The 857 metabolites were divided into 12 classes: phenolic acids; lipids; flavonoids; amino acids and derivatives; organic acids; nucleotides and derivatives; terpenoids; lignans and coumarins; and alkaloids, tannins, steroids, and others. Under drought stress, each class of metabolites varied slightly (Supplementary Figs. 1, 2, and 3), with the contents of triterpenoid saponins ranging more than the contents of the other classes. Furthermore, the total saponin levels of *E. senticosus* at 30–90% water content and 5-AzaC treatment were, in order, 14.38, 17.48, 8.71, 16.34, and 18.59 µg/g, according to the findings of the examination of the saponin content of *E. senticosus* (Fig. 2B). *E. senticosus* treated with 5-AzaC had the highest total saponin level; *E. senticosus* treated with 50% water content had the second-highest total saponin content. On the other hand, at 70% water content, *E. senticosus* had a significantly lower saponin level (*P* < 0.05).

A total of 44 metabolites were assessed to be significantly different in all treatment samples from *E. senticosus* under various drought conditions based on the|log^2^ fold change| ≥1 and VIP ≥ 1 criteria (Supplementary Table 2). A total of 239 differential metabolites were screened from the 50% water content treatment group with the highest genomic DNA methylation ratio and the lowest 5-AzaC treatment group of *E. senticosus*; 239 differential metabolites were screened (Fig. 2C). These metabolites were divided into eight categories: nucleotides and derivatives, amino acids and derivatives, phenolic acids, organic acids, lipids, flavonoids, lignans and coumarins, and others. Terpenoids are comprised of 21 different chemicals. The 5-AzaC treatment group significantly enriched 10 of these 21 compounds, pomolic acid and saponin PE, a total of 11 terpenoids, such as HN-saponin F and hederacoside, were significantly increased in the 50% water content-treated *E. senticosus* (Fig. 2D).

**Fig. 3:**
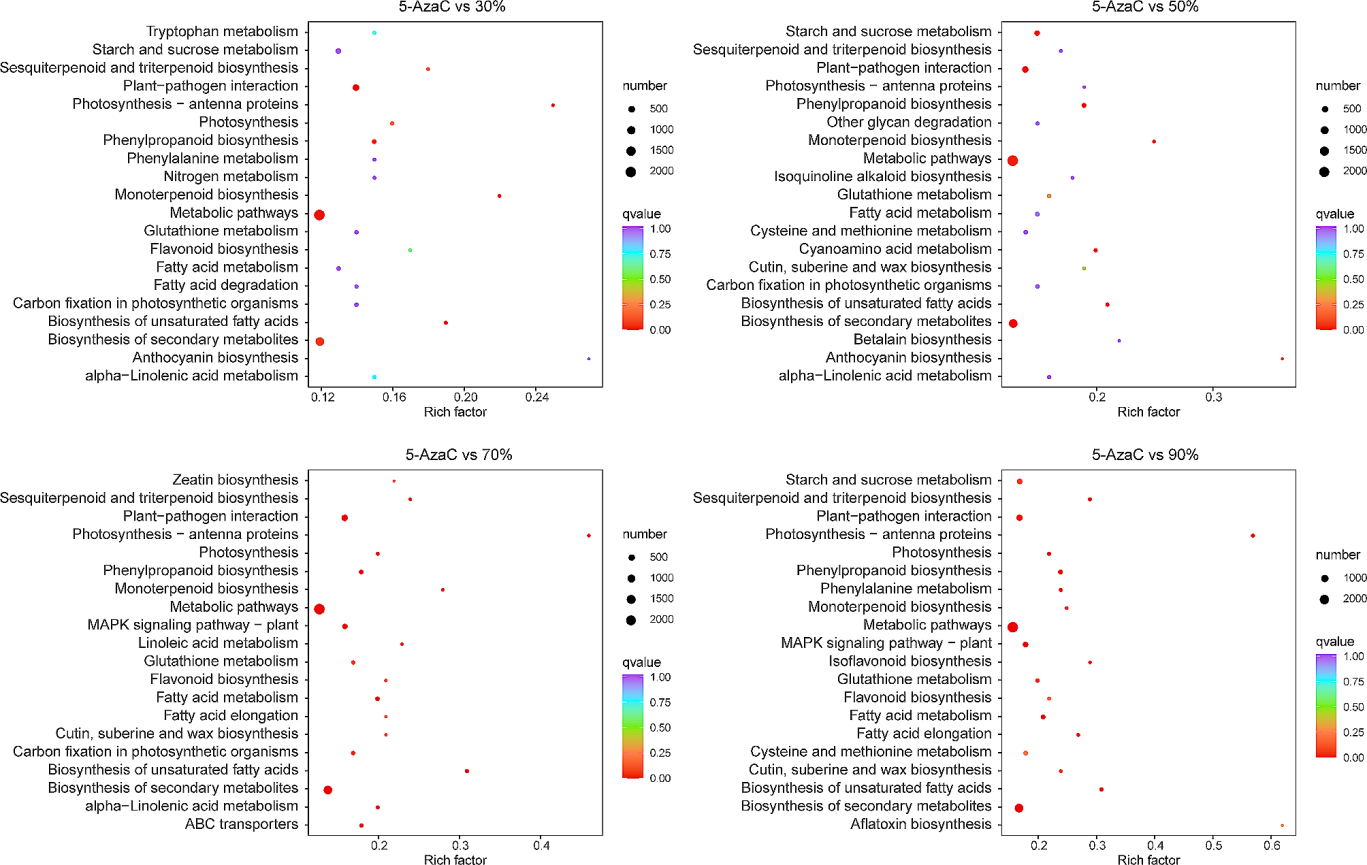
Analysis of Kyoto Encyclopedia of Genes and Genomics-enriched pathways of differential metabolites of *E. senticosus* under drought stress

The “metabolism” pathways of 30% vs 5-AzaC, 70% vs 5-AzaC, and 90% vs 5-AzaC were predominantly enriched in the following pathways, according to KEGG enrichment analysis of the differential metabolites (Fig. 3, Supplementary Fig. 4). “Phenylalanine metabolism,” “flavonoid biosynthesis,” and “flavonoid and flavonol biosynthesis.” Moreover, “linoleic acid” and “glucoside biosynthesis” were more abundant in 50% vs 5-AzaC. Enrichment in the “phosphatidylinositol signaling system” and “ABC transporter protein” were found in the comparable pathways of metabolite enrichment for intergroup variations in each group in “environmental information processing.” In the “genetic information processing” pathway, the differential metabolite enrichment pathways across the four comparison groups were the same. “Aminoacyl tRNA biosynthesis” was what they were all called. Four terpene metabolites were screened, and the 30% vs 5-AzaC screen revealed downregulated expressions of these metabolites; the 70% vs 5-AzaC screen revealed two downregulated and seven upregulated metabolites; the 90% vs5-AzaC screen revealed six downregulated and 10 upregulated metabolites. By comparison, when 21 terpenoid metabolites were screened in 50% vs 5-AzaC, the expression 11 was downregulated, and 10 were upregulated.

### Effect of drought stress on the transcription of *E. senticosus*

Transcriptome sequencing (Accession: SRX13417593-SRX13417601) of 30–90% water content and 5-AzaC-treated *E. senticosus* was performed using the Illumina HiSeq high-throughput sequencing platform. After splice-containing and low-quality reads were eliminated, 42.3–54.1 M clean reads with a Q30% of > 92% and GC contents of around 43% were obtained. After filtering the transcripts from Trinity splicing, 253, 490, and 244, 220 unigenes were obtained. The N50 was 1,371 bp, and the total length of unigenes was 107, 119, and 884 bp, with an average sequence length of 897 bp. The sequence lengths of unigenes between 200 bp and 1 000 bp were 172, 580 (70.67%), and 71, 636 (22.93%). The unigene sequences were compared with the KEGG, NR, SwissProt, GO, COG/KOG, and Trembl databases using DIAMOND BLASTX software (Supplementary Tables 3 and Supplementary Fig. 5). Following the prediction of the unigene amino acid sequences, the unigene annotation was obtained by comparing the unigene data with the Pfam database using the HMMER software.

**Fig. 4:**
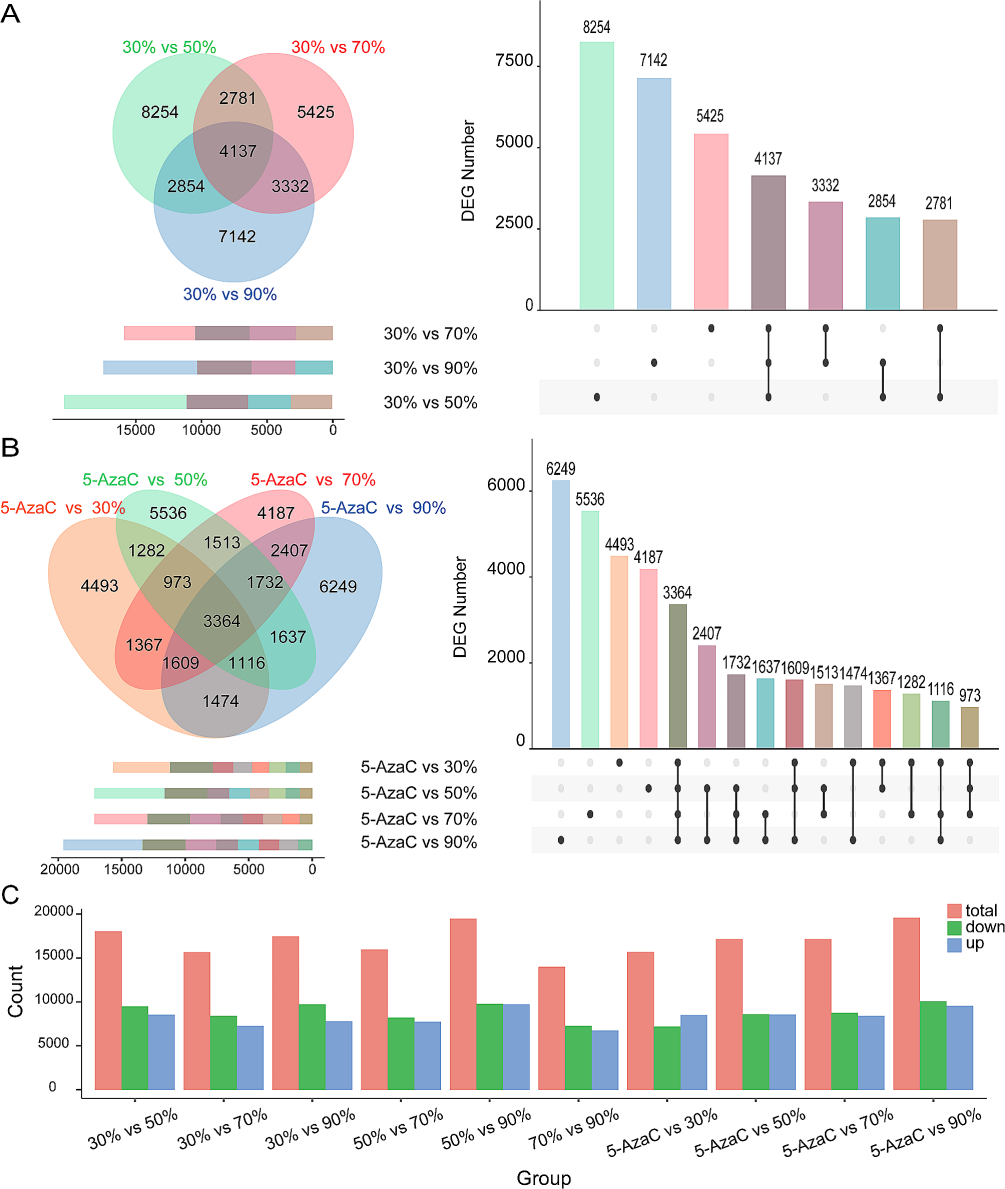
*E. senticosus* gene expression changes under drought stress. **A**: *E. senticosus* differential genes in treatments with 30–90% water content; **B**: *E. senticosus* differential genes in treatments with 30%–90% water content and 5-AzaC; and **C**: Upregulation and downregulation of differential genes among *E. senticosus* groups under various drought stressors

From the transcriptome sequencing data of 30%–90% water content-treated *E. senticosus*, a total of 33,925 differentially expressed genes were screened using FDR < 0.01 and|log^2^ FC| ≥1 as the screening criteria (Fig. 4A, Supplementary Figs. 6, 7, 8, 9). Of these, 4,137 genes exhibited varying differential expression levels in response to drought stress (Fig. 4A, B). From the transcriptome sequencing data of 30–90% water content vs 5-AzaC treatment of *E. senticosus*, 51,481 differentially expressed genes were screened (Fig. 4B, Supplementary Fig. 6). The effects of drought-induced DNA methylation on the expression changes of these genes varied. Of them, when the moisture conditions were the same, but the DNA methylation status was different, 3,364 genes in the 50% vs 5-AzaC comparison group exhibited significant changes in expression (Fig. 4C, Supplementary Fig. 6). Regardless of changes in water content when comparing with the 5-AzaC treatment group, the results of the KEGG enrichment pathway analysis of these 51,481 genes that were differentially expressed due to DNA methylation showed (Supplementary Fig. 10) that the differentially expressed genes were significantly enriched to biosynthetic pathways of secondary metabolites, such as triterpene saponins, phenylpropanes, and others. However, the secondary metabolite biosynthesis pathways of the 50% vs 5-AzaC-treated, which include triterpenoid saponins, were significantly enriched.

### Effect of drought stress on the transcription of the saponin synthase gene of *E. senticosus*

**Fig. 5:**
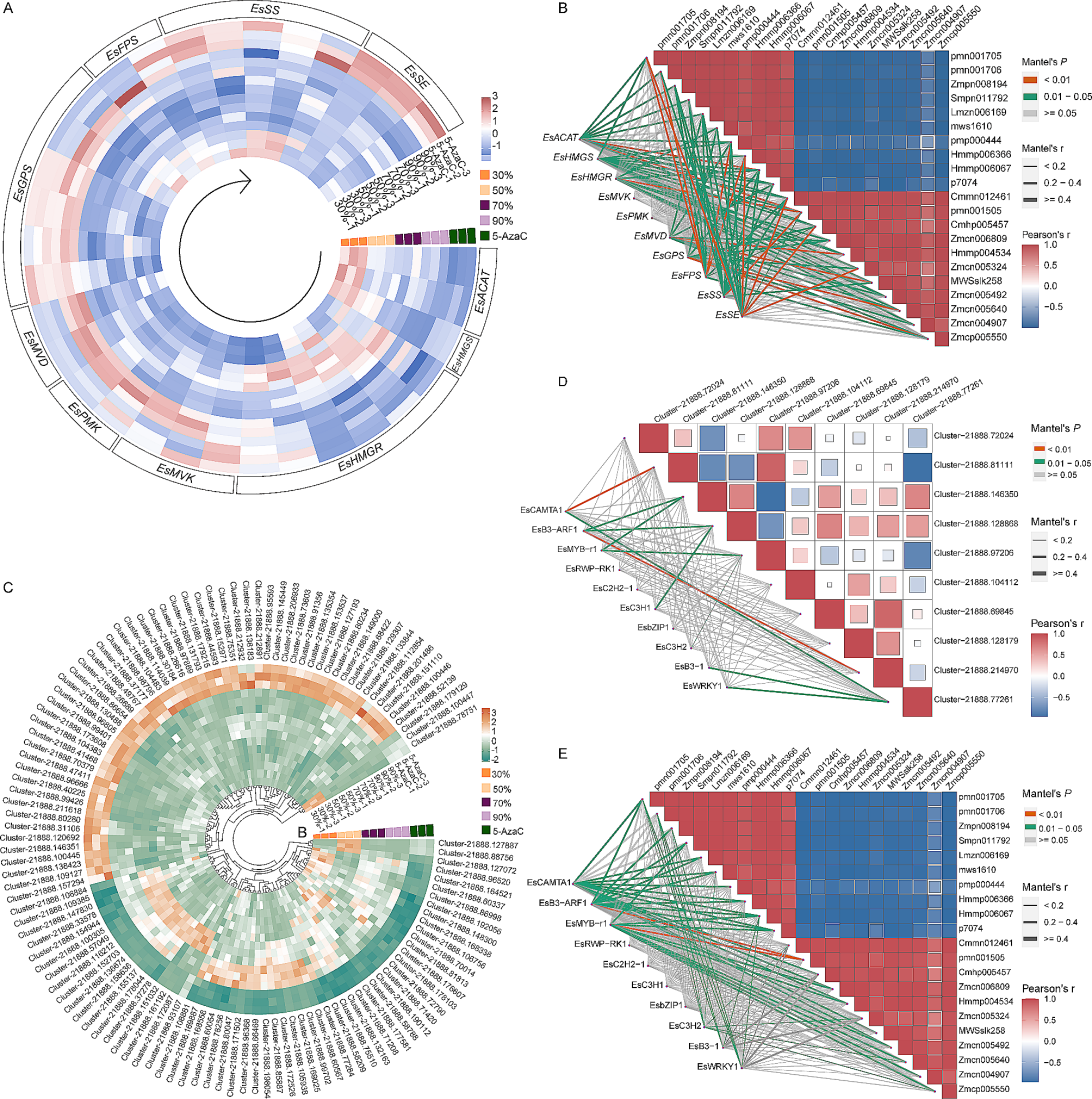
Screening of differentially expressed saponin synthesis genes and transcription factors in *E. senticosus* under drought stress. **A**: The expression level of triterpenoid saponin synthase gene; **B**: The correlation between differentially expressed genes in the synthesis pathway of triterpenoid saponins and terpenoid metabolites; **C**: Differential expression of transcription factors; **D**: The correlation between differentially expressed transcription factors and *EsFPS*, *EsSS*, and *EsSE* genes; **E**: The correlation between differentially expressed transcription factors and the content of triterpenoid saponins. Note: Pmn001705: 3,24-Dihydroxy-17,21-semiacetal-12(13)oleanolic fruit; pmn001706: 2-hydroxyoleanolic acid; Zmpn008194: Corosolic acid; Smpn011792: 2,3-dihydroxy-12-ursen-28-oic acid; Lmzn006169: Pomolic acid; mws1610: Maslinic acid; pmp000444: 27,28-Dicarboxyl ursolic acid; Hmmp006366: Saponin PE; Hmmp006067: Oleanolic acid-3-O-glucosyl(1→2)glucoside; p7074: ciwujianosidesD2; Cmmn012461: Dehydroabietic acid; pmn001505: Oleanolic acid-3-O-xylosyl(1→3)glucuronide; Cmhp005457: HN-saponin F; Zmcn006809: Oleanolic acid-3-O-rha(1–2)-(ara)-28-O-glucoside ester; Hmmp004534: Hederagenin-3-O-glucosyl(1–2) glucosyl(1–4)arabinoside; Zmcn005324: Ciwujianoside C3; MWSslk258: Hederacoside D; Zmcn005492: 3-O-Ara-Oleanolic acid-28-O-glc-glc(-Ac)-rha; Zmcn005640: Ciwujianoside D1; Zmcn004907: 3-O-Ara-rha-Oleanolic acid-28-O-glc-glc-rha-30-Methoxy; and Zmcp005550: hederagenin-3-O-glc-(1–4)[L-rha(1–3)]D-glc(1–3)L-rha(1–2)L-ara. Cluster-21888.72024 and Cluster-21888.81111: *EsFPS*; Cluster-21888.146350, Cluster-21888.128868, Cluster-21888.97206, Cluster-21888.104112, and Cluster-21888.69845: *EsSS*; Cluster-21888.128179, Cluster-21888.214970, and Cluster-21888.77261: *EsSE*

The expression of 10 different enzyme gene families (Fig. 5A, Supplementary Table 4) was examined concerning the cascade of catalytic saponin biosynthesis in *E. senticosus*. These include cholesterol acyltransferases (*EsACAT*), 3-hydroxy-3-methylglutaryl-CoA synthase (*EsHMGS*), 3-hydroxy-3-methylglutaryl-CoA reductase (*EsHMGR*), mevalonate kinase (*EsMVK*), phosphomevalonate kinase (*EsPMK*), mevalonate bisphosphate decarboxylase (*EsMVD*), geranyl pyrophosphate synthase (*EsGPS*), *EsFPS*, *EsSS*, and *EsSE*. It was discovered that following changes in the DNA methylation status of *E. senticosus* caused by drought stress, the expression of the 10 saponin synthase genes was modified to varying degrees. Among them, 5-AzaC-treated *E. senticosus* showed an overall upregulation of more than 1-fold in the expressions of *EsFPS*, *EsSS*, and *EsSE* compared to 50% water stress. The ratios of DNA methylation at the promoters of *EsFPS*, *EsSS*, and *EsSE* genes and the genomic DNA methylation were significantly reduced following 5-AzaC treatment.

The expression of the aforementioned 10 *E. senticosus* saponin synthase genes was correlated with the amount of differentially accumulated terpenoids (Fig. 5B) to screen out further the crucial genes for drought stress causing changes in the DNA methylation level of *E. senticosus* and consequently the saponin content. The findings demonstrated a positive correlation between the elevated expression of *EsFPS*, *EsSS*, and *EsSE* at low methylation levels and all saponins with increased contents that reached the *P* < 0.05 level. Similarly, the expression levels of the *EsFPS*, *EsSS*, and *EsSE* genes positively correlated with the contents of 4, 1, and 4 saponins at the *P* < 0.01 level, respectively. According to this, the essential genes that modify saponin synthesis and accumulation in *E. senticosus* caused by drought stress are *EsFPS*, *EsSS*, and *EsSE*. Simultaneously, the correlation between the promoter methylation ratio and gene expression of *EsFPS*, *EsSS*, and *EsSE* was analyzed using the Pearson correlation calculation method. Under varying water contents, the promoter methylation and expression of *EsFPS*, *EsSS*, and *EsSE* showed varying degrees of negative correlation patterns, with correlations of -0.83, -0.91, and − 0.85, respectively. These results suggest that the higher the promoter methylation ratio, the lower the gene expression.

### Screening of differentially expressed transcription factors of *E. senticosus* under drought stress

A total of 119 significantly differentially expressed transcription factors were screened using|log^2^ FC| ≥1 as the criterion from the transcriptome sequencing data of 30%–90% water content and 5-AzaC-treated *E. senticosus* (Fig. 5C, Supplementary Fig. 11). These transcription factors were then categorized into a total of 36 classes (Supplementary Table 5). Among these, a negative correlation was seen between the upregulation of 60 transcription factors and the increase in the DNA methylation ratio. A positive correlation was seen between the increase in DNA methylation ratio and the upregulation of 59 transcription factors.

After drought stress altered the DNA methylation level of *E. senticosus*, we further screened out potential crucial transcription factors that regulate changes in the expression of essential enzyme genes for saponin synthesis in *E. senticosus*. Specifically, we screened out the most significant differences in the expression of *Cluster-21888.100305* (named: *EsCAMTA1*), *Cluster-21888.66469* (named: *EsB3-ARF1*), *Cluster-21888.100446* (named: *EsMYB-r1*), *Cluster-21888.72858* (named: *EsRWP-RK1*), *Cluster-21888.184553* (named: *EsC2H2-1), Cluster-21888.184553* (named: *EsC2H2-1)*, *Cluster-21888.113627* (named: *EsC3H1*), *Cluster-21888.108881* (named: *EsbZIP-1*), *Cluster-21888.202629* (named: *EsWRKY1*), and *Cluster-21888.161192* (named: *EsWRKY2*). The results indicated (Fig. 5D) that the nine transcription factors had varying degrees of correlation with the expression of the *EsFPS*, *EsSS*, and *EsSE* genes. The transcription mentioned above factors were correlated with the expression of *essential* enzyme genes for spiny saponin synthesis, respectively. The expressions of most of the *EsFPS*, *EsSS*, and *EsSE* genes and the expressions of *EsMYB-r1*, *EsB3-ARF1*, and *EsCAMTA1* (Supplementary Table 6) among them show significant positive correlations (*P* < 0.05), with the correlations being significantly higher than those of other transcription factors. Each transcription factor typically correlated with triterpenoid saponins to varying degrees, according to further correlation analysis of the nine transcription factors with 21 triterpenoid saponin differential metabolites revealed (Fig. 5E). *EsMYB-r1*, *EsB3-ARF1*, and *EsCAMTA1* showed the highest correlation with triterpene saponin differential metabolites among all transcription factors, consistent with the expression correlation data. There was a significant and positive correlation (*P* < 0.05) between *EsMYB-r1*, *EsB3-ARF1*, and *EsCAMTA1* and 9, 8, and 11 triterpene saponin differential metabolites, respectively (*P* < 0.05). Furthermore, the *P* < 0.01 level was attained by the positive correlations between *EsB3-ARF1*, *EsMYB-r1*, and Oleanolic acid-3-O-xylosyl(1→3)glucuronide. This suggested that the transcription factors *Es*MYB-r1, *Es*B3-ARF1, and *Es*CAMTA1 may act as a bridge and link to transmit the DNA methylation information to the crucial enzyme genes for saponin synthesis, *EsFPS*, *EsSS*, and *EsSE*, and alter the expression of these genes, which in turn leads to the synthesis of triterpenoids changes in the synthesis after drought stress-induced changes in the DNA methylation status of *E. senticosus*.

### Subcellular localization of crucial transcription factors

**Fig. 6:**
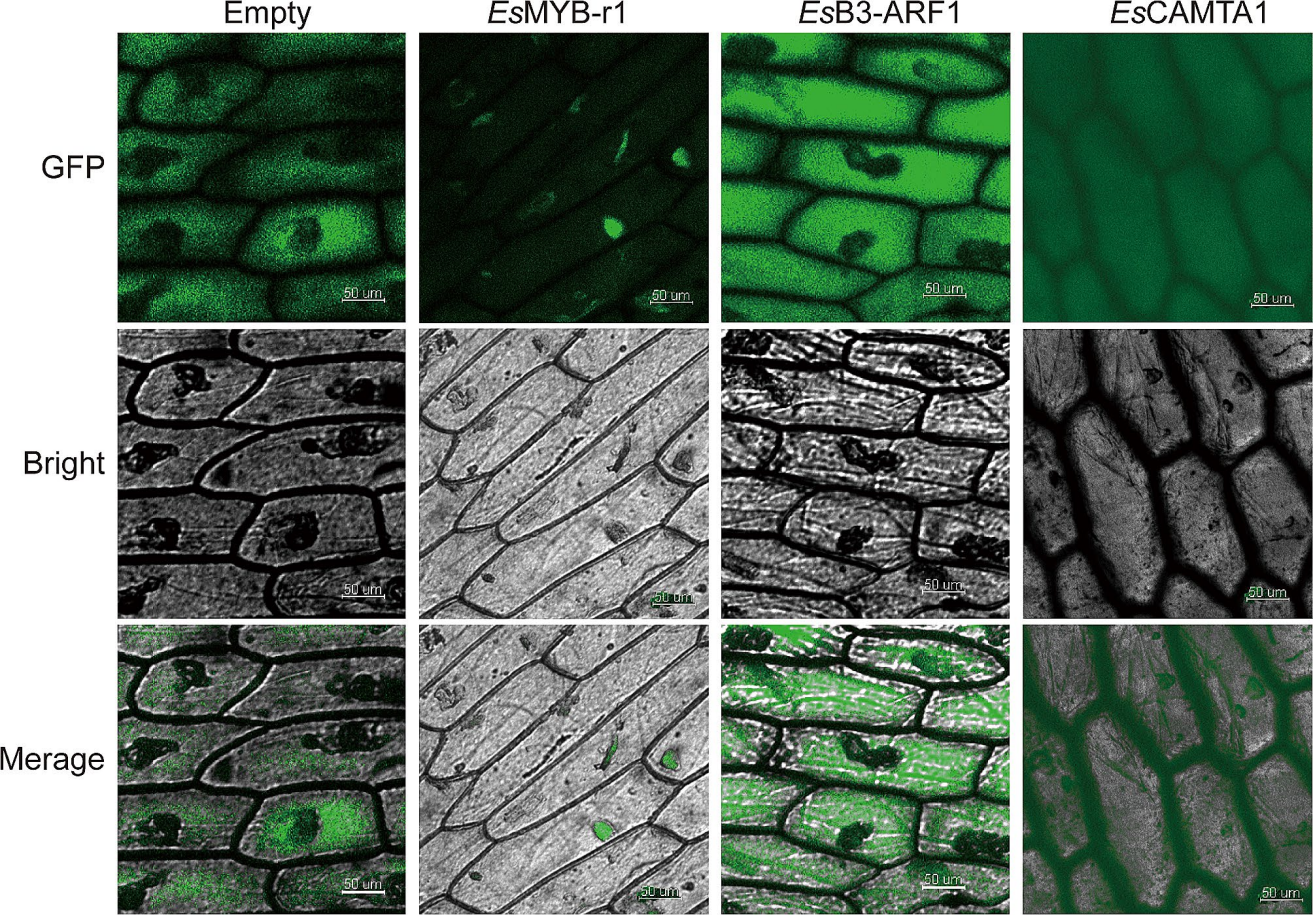
Subcellular localization of crucial transcription factors of *E. senticosus* under drought stress

The WoLF PSORT website predicted the subcellular localization of the three transcription factors that exhibited the highest correlation with the expression of *EsFPS*, *EsSS*, and *EsSE* genes. The results indicated that *Es*MYB-r1, *Es*B3-ARF1, and *Es*CAMTA1 localized in the nucleus or cytoplasm. *Es*MYB-r1, *Es*B3-ARF1, and *Es*CAMTA1 were fused with GFP and transiently expressed in the *A.cepa* epidermis. As a result, *Es*MYB-r1 was localized in the nucleus, while *Es*B3-ARF1 and *Es*CAMTA1 were localized in the cytoplasm (Fig. 6).

### Analysis of the binding capacity of crucial transcription factors to *EsFPS*, *EsSS*, and *EsSE* promoters

**Fig. 7:**
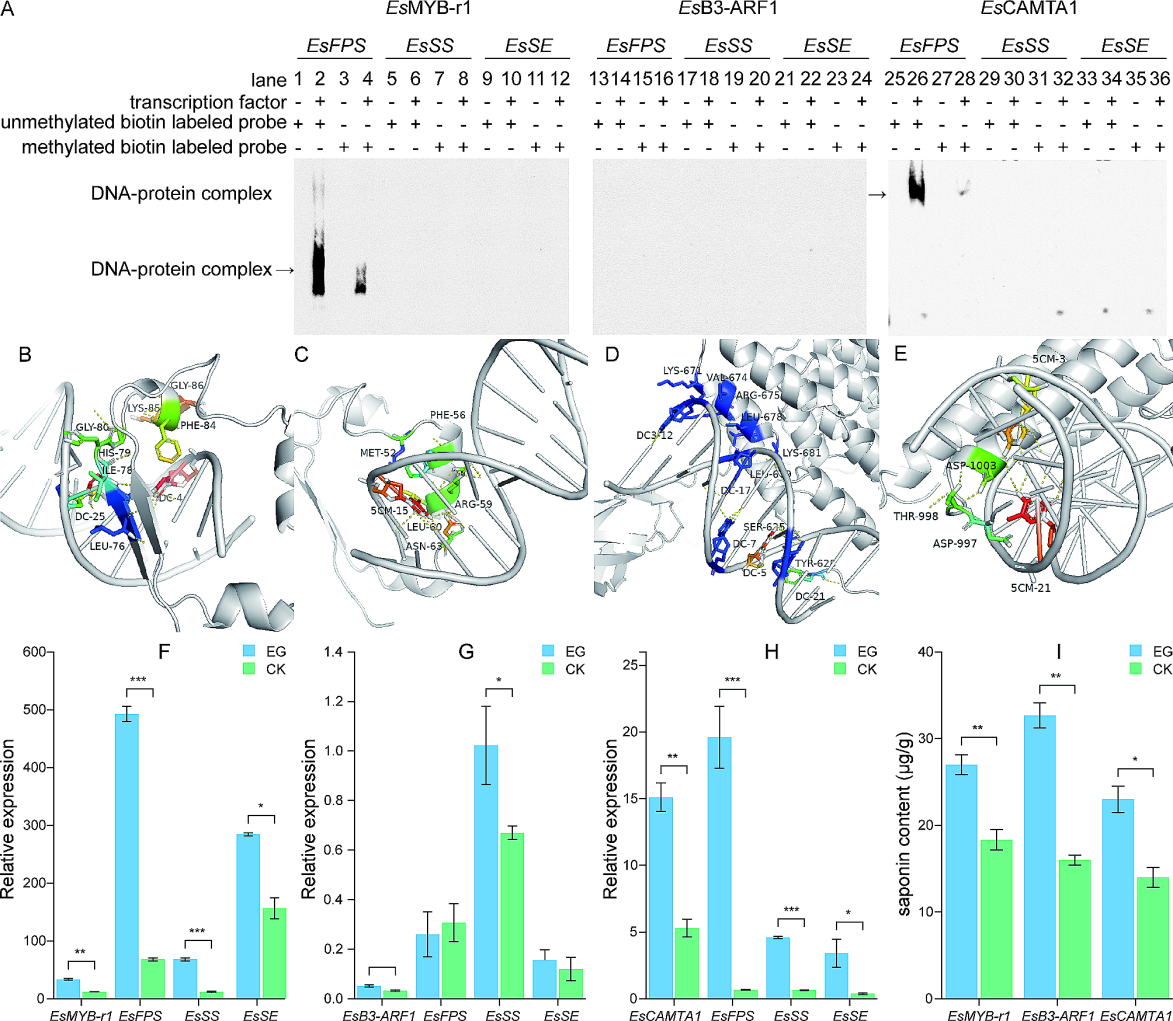
Binding power and overexpression analysis of key transcription factors with *EsFPS*, *EsSS*, and *EsSE* promoters. **A**: Electrophoretic mobility shift assay analysis of crucial transcription factor binding to *EsFPS*, *EsSS*, and *EsSE* promoters; **B**: Molecular docking of *Es*MYB-r1 with unmethylated *EsFPS*; **C**: Molecular docking of *Es*MYB-r1 with methylated *EsFPS*; **D**: Molecular docking of *Es*CAMTA1 with unmethylated *EsFPS*; **E**: Molecular docking of *Es*CAMTA1 with methylated *EsFPS*; **F**: Gene expression after *EsMYB-r1* overexpression; **G**: Gene expression following *EsB3-ARF1* overexpression; **H**: Gene expression following *EsCAMTA1* overexpression; and **I**: triterpene saponin content following overexpression of sensitive transcription factor genes. *P ≤ 0.05, **P ≤ 0.01, and ***P ≤ 0.001. Note: 1–4, 13–16, and 25–28: promoters of *EsFPS*; 5–8, 17–20, and 29–32: promoters of *EsSS*; 9–12, 21–24, and 33–26: promoters of *EsSE*

EMSA analysis was performed on the in vitro expressed and purified *Es*MYB-r1, *Es*B3-ARF1, and *Es*CAMTA1 using probes of biotin-labeled *EsFPS*, *EsSS*, and *EsSE* promoters, respectively. The findings demonstrated (Fig. 7A) that the unmethylated *EsFPS* promoter could be bound by *Es*MYB-r1 and *Es*CAMTA1, forming a clear blocking band at 36 kDa and 134 kDa, respectively. In contrast, in any condition, *Es*MYB-r1 and *Es*CAMTA1 could not bind to the *EsSS* and *EsSE* promoters. Additionally, following DNA methylation of the *EsFPS* promoter, the blocking bands significantly lightened, suggesting that DNA methylation inhibited the binding of *Es*MYB-r1 and *Es*CAMTA1 to the *EsFPS* promoter. However, regardless of whether DNA methylation occurred in these promoters, *Es*B3-ARF1 was unable to produce blocking bands with *EsFPS*, *EsSS*, and *EsSE* promoters, indicating that *Es*B3-ARF1 was not able to bind to these promoters directly.

### Molecular dynamic analysis of *EsFPS* promoter binding to *Es*MYB-r1 and *Es*CAMTA1

PDB models for molecular docking were created using the predicted binding sites of the transcription factor proteins *Es*MYB-r1 and *Es*CAMTA to the *EsFPS* promoter region, as determined by the JASPER website (Fig. 7B-E). Seven amino acid residues in the *E*sMYB-r1 protein, including GLY86, LYS85, PHE84, GLY80, HIS79, ILE78, and LEU76, were able to bind to the cytosine in the promoter region of *EsFPS* when *EsFPS* was not methylated (Fig. 7B). As a result, there was no spatial conflict between the amino acid residues of LYS85, which were neighboring to GLY86 and formed a groove structure spatially to surround the cytosine. The grooves formed between amino acid residues that were previously bound to cytosine were unable to adapt to methylated cytosine, which results in the fact that only MET52, PHE56, LEU60, ARG59, and ASN63 in the *Es*MYB-r1 protein are near methylcytosine. This is because the spatial conformation of methylcytosine was different from that of cytosine when the *EsFPS* promoter cytosine (Fig. 7C). The interaction between methylcytosine and weak hydrogen bonds caused a decrease in the binding of *Es*MYB-r1 protein to methylated *EsFPS*. This phenomenon was more evident in the binding of the *Es*CAMTA protein to the *EsFPS* promoter, where a total of eight amino acid residues, namely LYS671, VAL674, ARG675, LEU678, LYS681, LEU689, SER625, and TYR628, were able to bind to the cytosine of the *EsFPS* promoter (Fig. 7D). In contrast, when the cytosine of the *EsFPS* promoter is methylated (Fig. 7E), this groove does not adapt to the methylated cytosine, and only ASP1003, THR998, and ASP997 interact with the methylated cytosine by weak hydrogen bonding, resulting in a decrease in the binding strength of *Es*CAMTA to the methylated *EsFPS* promoter.

### Overexpression analysis of *EsMYB-r1*, *EsB3-ARF1*, and *EsCAMTA1*

*Es*MYB-r1, *Es*B3-ARF1, and *Es*CAMTA1 are essential transcription factors that we ligated into the pCAMBIA1300 overexpression vector to temporarily express these genes in *E. senticosus*. This allowed us to investigate the effects of these transcription factors on the synthesis of essential enzyme genes and secondary metabolites of saponin synthesis in *E. senticosus* saponins. Quantitative reverse transcription PCR analysis demonstrated that the expression of *Es*MYB-r1, *Es*B3-ARF1, and *Es*CAMTA1 was significantly higher than that of the control *E. senticosus* (Fig. 7F-H) (*P* < 0.01), suggesting that the overexpression of the essential transcription factors mentioned above in *E. senticosus* was successfully achieved. The overexpression of *Es*MYB-r1 and *Es*CAMTA1 significantly increased (*P* < 0.05) in the EsFPS, EsSS, and EsSE expressions. Of these, the expression of *EsFPS* was the most elevated, reaching 7.24-fold and 29.25-fold of the control group, respectively, and significantly higher than that of *EsSS* and *EsSE* (*P* < 0.05). Although none reached the significant level, overexpression of *Es*B3-ARF1 significantly increased the expression of *EsSS* (*P* < 0.01), somewhat decreased the expression of *EsFPS*, and marginally increased the expression of *EsSE*. The total saponin content assay findings (Fig. 7I) demonstrated that overexpressing *Es*MYB-r1, *Es*B3-ARF1, and *Es*CAMTA1 (*P* < 0.05) resulted in a significant increase in total saponin content, reaching 1.47-fold, 2.04-fold, and 1.64-fold of the control group, respectively.

## Discussion

A growing body of research indicates that modifications to the levels of secondary metabolites accumulated [29–30] and DNA methylation status of the genome and functional genes [31–32] are essential for plant adaptation to drought stress. Uncertainty exists regarding the precise method by which DNA methylation controls plant secondary metabolism during drought. Thus, it is imperative to explore the function of DNA methylation in regulating secondary metabolism and improving drought tolerance in plants. In this study, we investigated how variations in water conditions affected the DNA methylation status of the *E. senticosus* genome and the saponin synthase gene promoter. These modifications caused transcription factors to no longer bind to target genes, which changed the expression of those genes and saponin synthesis. These findings offer important new information about the epigenetic regulation mechanism of drought stress adaptation in plants.

### Effects of drought stress on the secondary metabolism of *E. senticosus*

Less water in the soil leads to drought stress, typically caused by insufficient rainfall and rising temperatures, which results in continual water loss through transpiration and evaporation [33]. Plant biosynthesis is altered, and secondary metabolites accumulate due to water deprivation [34]. Moderate drought stress is generally favorable for the synthesis and accumulation of secondary metabolites in plants, which is a manifestation of the adaptation of most plants to drought life [35]. Drought stress generally reduces plant growth and biomass accumulation [36].

The results of the analysis of 27 provenances of *E. senticosus* showed that precipitation was shown to be substantially and positively correlated with both the growth and development of *E. senticosus* and photosynthesis and negatively correlated with the amount of *E. senticosus* saponins present in the 27 provenances according to the findings of the analysis [37]. Additional studies on drought stress revealed that moderate drought stress was best for the accumulation of saponins in *E. senticosus* and that either sufficient water or severe water deficit showed a significant reduction in saponin synthesis in *E. senticosus.* Additionally *E. senticosus* biomass and photosynthesis consistently demonstrated a significant positive correlation with the soil water content [20]. This indicates that while moderate drought stress promotes the production of *E. senticosus* secondary metabolites, a moist soil condition is favorable for the plant’s development and photosynthesis. This is in line with the observation that *E. senticosus* at 50% water content (moderate drought stress) had substantially larger total saponin levels and most types of monomeric saponins than those at too much or too little water in this study. It is also in line with the observation that most medicinal plants exhibit an increase in secondary metabolite accumulation under mild or moderate drought stress [38] and that severe drought stress can negatively impact secondary metabolite synthesis and stunt plant growth [34, 35]. These plants had all of the same qualities.

The way that plants react to drought stress is a complicated process. Still, generally speaking, the build-up of active ingredients in medicinal plants is caused by changes in the related metabolism brought on by drought stress [39]. Drought stress is the root cause of the build-up of active substances in medicinal plants is primarily caused by drought stress. Specifically, stomata are closed, and CO_2_ uptake significantly decreases due to inadequate water supply [8]. This causes a significant decrease in the consumption of reducing equivalents (NADPH + H^+^), which is above demand due to the reduction of CO_2_ fixed by the Calvin cycle. This pushes metabolic processes toward synthesizing secondary metabolites like highly reduced saponin [34]. These abundant saponin compounds serve as antioxidants, assisting *E. senticosus* in eliminating stress on its cells by scavenging reactive oxygen species produced in the body due to oxidative stress brought on by drought stress [7]. Ultimately, it makes *E. senticosus* more tolerant of drought stress and allows it to survive in environments with limited water supply.

Drought stress affects the accumulation of distinct secondary metabolites differently, even though modest water scarcity can generally increase the total amount of secondary metabolites. For instance, the drought-stressed plants of *Mentha piperita* and *Catharanthus roseus* displayed declines in total phenols of 21.46% and 29.57%, flavonoids of 37.57% and 39.96%, and saponins of 17.95% and 66.20%, respectively. However, the stress of the drought also caused increases in tannins, alkaloids, and terpenoids in both species. The highest increases were observed in total phenols 29.14%, flavonoids 37.57% and 39.96%, and saponins 17.95% and 66.20%. Following exposure to the combined stress, the maximum increases were seen in *M. piperita* and *C. roseus* for tannins at 29.14% and 50.16%, alkaloids at 39.39% and 53.72%, and terpenoids at 6.59% and 36.11%, respectively [40]. Similar characteristics were shown in *E. senticosus*, wherein at 50% water content, the ideal level for saponin accumulation, some triterpene saponins were greatly increased, and others were significantly decreased. This is mainly because plants first produce secondary metabolic upstream chemicals with very modest molecular weights in response to water scarcity [31]. More -OH, -OCH_3,_ and unsaturated double bonds are present in these upstream compounds, which can directly and quickly increase the antioxidant capability of cells [41]. However, these upstream antioxidant active products become complex compounds like downstream saponins and are retained when drought stress continues [31]. Simultaneously, varying drought stress levels elicited distinct oxidative stress responses [29], leading to variations.

### Role of DNA methylation in response to drought stress in *E. senticosus*

Several secondary metabolites, like saponins, are found in medicinal plants, such as *E. senticosus*. These metabolites work together to help the plant resist environmental drought stress [41], mitigate its adverse effects, and increase its survival ability [42–43]. These secondary metabolites need to be catalyzed by several enzymes to form and accumulate these secondary metabolites [44]. Thus, the alteration in the expression levels of genes for the enzymes involved in the production of these secondary metabolites is directly responsible for the change in secondary metabolite content in medicinal plants during drought stress [45–47]. For instance, *G. glabra* increases gene expression, including 3-hydroxy-3-methyl-glutaryl-coenzyme A reductase, squalene synthase, and β-amyrin synthase [48]. Similarly, during drought stress, the expression of the genes involved in saponin, *EsFPS*, *EsSS*, and *EsSE* changed in *E. senticosus* to varying degrees. Most of these gene expression changes were positively correlated with changes in saponin content.

Plants respond to drought stress by modifying the accumulation of their secondary metabolites through a complicated network of gene expression levels [49]. Drought has been shown in recent years to affect the DNA methylation status of plants, and these modifications are site-specific and stress-specific [50]. Our study on *E. senticosus* further supported this theory, showing that the genomic DNA methylation ratios at 30% and 50% water content were significantly higher than those at 70%. Additionally, previous studies have shown that DNA methylation can significantly decrease the promoter regions of *EsFPS*, *EsSS*, and *EsSE*, the crucial enzymes for synthesizing triterpenoid saponin in *E. senticosus*. This, in turn, lowers the concentration of triterpenoid saponins in *E. senticosus* [18–19]. This suggests that *E. senticosus* uses DNA methylation as an essential epigenetic alteration to modify gene expression and the concentration of secondary metabolites in response to drought stress.

Interestingly, under drought stress, the triterpene saponin concentration, the *EsFPS, EsSS*, and *EsSE* promoters had the lowest DNA methylation ratios, whereas *E. senticosus*, with 50% water content, showed the highest genomic DNA methylation ratio. This implies that the DNA methylation of specific functional genes and general genomic DNA methylation were not always positively correlated. For instance, under drought stress, the intergenic, exon, intron, and downstream regions of the *Morus alba L.* genome have increased methylation levels. Nevertheless, the methylome level was reduced in utr3prime, utr5prime, splice site region, and splice site acceptor region of the genome and gene region [51]. Additionally, prior research has shown that DNA methylation is less prevalent in the gene and first exon sections of the genome and is mainly dispersed in the intergenic, exon, intron, downstream, and upstream regions [52]. Specific functional gene DNA methylation levels influence certain metabolic pathways significantly higher than total genomic DNA methylation levels [53–54]. This also explains why, at 50% water content, the promotor for the synthesis of saponins, namely *EsFPS*, *EsSS*, and *EsSE*, had the lowest rates of DNA methylation of any region of the genome. However, the whole genome was the highest. This led to the increased expression of genes involved in the saponin synthesis pathway and increased synthesis of saponins. In contrast, 5-AzaC-treated *E. senticosus* exhibited a higher concentration of triterpenoid saponins and a decreased DNA methylation ratio at the same water content. This is because 5-azaC, an analog of cytosine nucleoside, inhibits DNA methyltransferase [55]. Thus, 5-azaC significantly lowered the DNA methylation level of *E. senticosus*. Additionally, there is typically a negative correlation between the DNA methylation level and the saponin content of *E. senticosus* [18–19].

### **Transcription factor-mediated DNA methylation regulates gene expression and saponin synthesis to adapt** ***E. senticosus*** **during drought stress**

Several studies have demonstrated that the expression of downstream genes is significantly influenced by the level of DNA methylation at promoters in various organisms, including medicinal plants [53]. DNA methylation does not directly produce biological effects, such as the regulation of gene expression [56–57]. According to recent research, unmethylated DNA in *Arabidopsis thaliana* contains most WRKY transcription factors [58]. According to site-specific DAP-qPCR data, AtWRKY40 binding is found on unmethylated promoters [59] but not when tested using genomic DNA from demethylase gene mutants [58]. According to additional research, a spatial barrier between AtWRKY40 and the conserved tyrosine of the DNA binding domain is created when a single cytosine in the promoter transcription factor binding site (TFBS) is methylated. Ultimately, this prevents *At*WRKY40 from attaching to methylated DNA [60].

Here, we present the crystal structures of the molecular docking of the *EsFPS* promoter with specific structural domains of *Es*MYB-r1 and *Es*CAMTA1, together with an explanation of the molecular characteristics of the nucleotides and amino acids at the binding site. *Es*MYB-r1 and *Es*CAMTA1 engage in hydrogen bonding with the unmethylated *EsFPS* promoter. While methylation cytosines change the 3D structure of the *EsFPS* promoter [61], their hydrophobic methyl groups directly prevent the *Es*MYB-r1 and *Es*CAMTA1 grooves to the target DNA from being close to it, forming a barrier to binding [62]. A binding barrier is formed when the hydrophobic methyl groups in methylated cytosines directly prevent the target DNA from coming into close contact with the *Es*MYB-r1 and *Es*CAMTA1 grooves. As a result, methylated *EsFPS* distances itself from the amino acid residues of *Es*MYB-r1 and *Es*CAMTA1, making it more challenging to form sufficient hydrogen bonds. Ultimately, this prevents the transcription factor from binding to the target DNA. This is consistent with the fact that the binding of R2R3-MYB-type transcription factors to methylated target DNA in humans is reduced by more than 45-fold compared to unmethylated DNA [48]. Therefore, the regulation of gene expression by DNA methylation depends on altering the binding of transcription factors to target DNA [63].

In this study, *Es*MYB-r1, *Es*B3-ARF1, and *Es*CAMTA1 were all significantly correlated with the expression of essential enzyme genes (*EsFPS*, *EsSS* and *EsSE*) for *E. senticosus* saponin synthesis and the saponin content (*P* < 0.05). After overexpression, they differentially increased the expression level and saponin content of *EsFPS*, *EsSS*, and *EsSE.* However, the *EsFPS* promotor could only be bound by *Es*MYB-r1 and *Es*CAMTA1. This could be because of two reasons. First, the binding of *Es*MYB-r1 and *Es*CAMTA1 to the *EsFPS* promoter significantly increased the expression of the *EsFPS* gene, increasing farnesyl diphosphate [64]. This excess farnesyl diphosphate then functioned as a substrate for the catalysis of *EsSS*, resulting in high expression of *EsSS* [65]. Similarly, the expression levels of following saponin synthesis-related enzyme genes were progressively elevated, which eventually resulted in a simultaneous increase in the saponin content of *E. senticosus* and enhanced the ability of *E. senticosus* to adapt to drought stress. Second, although *Es*B3-ARF1 cannot bind to the promotors of the genes of *EsFPS*, *EsSS*, and *EsSE*, it may bind to the promoters of the genes of other enzymes involved in the synthesis of saponins, producing an outcome similar to the first scenario. It has relatively minimal influence on the following processes if it binds the promoters of the enzyme genes that catalyze them: *EsFPS*, *EsSS*, and *EsSE* [66].

In this work, we unequivocally demonstrate that drought stress can modify the DNA methylation status of particular genes and the entire genome of *E. senticosus*. Under moderate drought stress, the methylation rate was lowered by the promoter of the primary enzyme genes for methylated saponin synthesis. This allowed demethylated cytosines to bind to methylation-sensitive transcription factors, upregulating gene expression. In *E. senticosus*, there was an increase in the manufacture and accumulation of saponin analogs due to elevated expression levels of genes encoding necessary saponin manufacturing enzymes. Because saponins function as antioxidants, they enhance *E. senticosus’s* ability to adapt to drought stress and live in settings lacking in water.

Given that *E. senticosus* has a large number of enzyme genes involved in the synthesizing of saponins and that it produces secondary metabolites other than saponins, additional transcription factors and secondary metabolites may play a role in the ability of *E. senticosus* to adapt to drought stress. Subsequent research on additional transcription factors and metabolites is necessary to provide a more thorough explanation of this regulation process, as this work only focuses on the three transcription factors that significantly impact saponin production.

### Electronic supplementary material

Below is the link to the electronic supplementary material.

Supplementary Material 1

Supplementary Material 2

Supplementary Material 3

Supplementary Material 4

Supplementary Material 5

Supplementary Material 6

Supplementary Material 7

## Data Availability

The datasets generated and/or analysed during the current study are available in the supplementary files. The raw datasets generated during the sequencing of current study are available in NCBI (Accession: SRX13417593-SRX13417601).

## Abbreviations

CDS: Coding DNA sequence
qRT-PCR: Quantitative real-time PCR
HPLC: High performance liquid chromatography
UPLC: Ultra performance liquid chromatography
5-AzaC: 5-Azacytidine
*EsFPS*: Farnesyl diphosphate synthase
*EsSS*: Squalene synthase
*EsSE*: Squalene epoxidase
EMSA: Electrophoretic mobility shift assay
