# Supplementary material for: DNA methylation regulates the secondary metabolism of saponins to improve the adaptability of Eleutherococcus senticosus during drought stress
Source: BMC Genomics. 2024 Apr 2;25:330. doi: 10.1186/s12864-024-10237-x (PMC10986080; doi:10.1186/s12864-024-10237-x)
Supplement: Supplementary file 1 — Supplementary Material 1 [file 12864_2024_10237_MOESM1_ESM.docx]

Supplementary Fig. 1 Differential metabolites clustering heat map

Supplementary Fig. 2 Differential metabolites radar map

Supplementary Fig. 3 Differential metabolites chord diagram

Supplementary Fig. 4 Differential metabolites clustering heatmap of KEGG pathway

A: 5-AzaC vs 30%; B: 5-AzaC vs 70%; C: 5-AzaC vs 90%; D: 30% vs 50%; E: 30% vs 70%; F: 30% vs 90%; G: 50% vs 70%; H: 50% vs 90%; I: 70% vs 90%.

Supplementary Fig. 5 NR, GO, KOG annotation of the transcriptome of *Eleutherococcus senticosus*

Supplementary Fig. 6 Differential genes clustering heatmap

Supplementary Fig. 7 KEGG enrichment scatter plot

Supplementary Fig. 8 Differential genes GO enrichment column chart

Supplementary Fig. 9 KOG classification column chart

Supplementary Fig. 10 Kyoto Encyclopedia of Genes and Genomics enrichment pathway analysis of *E. senticosus* differentially expressed genes under drought stress.

Supplementary Fig. 11 Transcription factors annotation classification statistical pie chart
